# Urban-rural differences in the incompleteness of antenatal care coverage in Indonesia: A cross-sectional study

**DOI:** 10.51866/oa.616

**Published:** 2025-03-20

**Authors:** Haerawati Idris, Rinda Nurul Karimah, Anni Yulianti

**Affiliations:** 1 PhD, Department of Health Policy and Administration, Faculty of Public Health, Sriwijaya University, Indralaya, Ogan Ilir, South Sumatera, Indonesia. Email: haera@fkm.unsri.ac.id; 2 PhD, Health Department, Politeknik Negeri Jember, East Java, Indonesia; 3 PhD, Health Department, Politeknik Negeri Jember, East Java, Indonesia

**Keywords:** Female, Pregnancy, Indonesia, Antenatal care

## Abstract

**Introduction:**

The estimated maternal mortality ratio in Indonesia from 2016 to 2020 was 249 maternal deaths per 100,000 live births. Currently, this ratio remains relatively high. One effort to reduce maternal mortality is to provide regular antenatal care during pregnancy. This study aimed to analyse the urban-rural differences in the incompleteness of antenatal care coverage in Indonesia.

**Methods:**

This cross-sectional study used data from Indonesian Basic Health Research 2018. A total of 64,399 women aged 15-49 years, including 26,792 and 37,607 women from urban and rural areas, respectively, were included. Univariate (percentage), bivariate (chi-square statistics) and multivariate (logistic regression statistics) analyses were conducted.

**Results:**

Approximately 18.2% and 26.4% of the urban and rural participants received incomplete antenatal care, respectively. Secondary and primary education, lack of health insurance, home-based antenatal care, parity greater than 2, travel time to health facilities exceeding 15 min, absence of abortion history, undesired pregnancy and absence of pregnancy complications were associated with incomplete antenatal care in rural areas. Secondary and primary education, home-based antenatal care, travel time to health facilities exceeding 15 min, parity greater than 2 and undesired pregnancy were associated with incomplete antenatal care in urban areas.

**Conclusion:**

Incomplete antenatal care coverage is more prevalent in rural areas than in urban areas, influenced by distinct socio-demographic and healthcare access factors. Strengthening health insurance programmes, improving healthcare facility access and promoting antenatal care education are critical to reducing disparities and ensuring better maternal health outcomes in both urban and rural areas.

## Introduction

Antenatal care (ANC) is crucial for enhancing maternal and child health by providing essential medical services, education, and support throughout pregnancy. Regular checkups help monitor maternal health, identify risk factors, and manage conditions such as hypertension and diabetes, which can adversely affect pregnancy outcomes.^1^ It is crucial as it promotes a healthy gestational period, supports expectant mothers, and improves perinatal and neonatal outcomes.^2^ and also for enhancing maternal and child health as it ensures the wellbeing of pregnant women and their unborn children, providing essential health services that can prevent complications and improve health outcomes during pregnancy and childbirth.^3^

There are some consequences of inadequate ANC. Women without ANC are at a higher risk of complications, including emergency caesarean sections and low-birth-weight babies.^4^ The maternal mortality ratio is significantly higher among unbooked pregnancies, underscoring the need for regular ANC engagement.^4^ ANC coverage varies significantly globally, particularly between low- and middle-income countries (LMICs) and higher-income regions. An analysis of 63 national surveys from LMICs indicated that socio-economic factors heavily influenced ANC access, with urban, educated and wealthier women experiencing higher coverage rates. The average ANC coverage in countries with high maternal mortality was approximately 55.66%, highlighting substantial gaps in service utilisation.^5,6^

Indonesia’s commitment to MCH is evident through various programmes aligned with the Sustainable Development Goals (SDGs), particularly Goal 3, which aims to reduce maternal and child mortality. The country has implemented strategies focusing on enhancing women’s autonomy, improving healthcare accessibility and integrating planning and budgeting to ensure effective service delivery. A study reported that higher autonomy among women correlated with increased utilisation of MCH services, with only 22.14% of mothers using all services continuously.^7^ The Indonesian government aims for at least four ANC visits per pregnancy, a key indicator for universal health coverage and maternal health outcomes outlined in the SDGs.^8^ Among national targets for ANC, one target is for all pregnant women to receive at least four ANC visits, which is crucial for monitoring maternal and foetal health.^8^ Recent studies have indicated that approximately 79.1% of women in rural areas receive at least four visits, but only 47.1% achieve the recommended eight visits.^9^

Urban-rural disparities in ANC services are influenced by a variety of socio-economic,^10,11^ educational^10^ and infrastructural factors.^8,11^ Despite numerous studies highlighting ANC utilisation in Indonesia, research addressing urban-rural disparities in incomplete ANC coverage still needs to be improved. Most existing studies focus on general coverage rates or regional comparisons without delving into the nuanced factors driving the incompleteness of ANC, particularly in rural areas where access to healthcare facilities and skilled providers is often constrained. Furthermore, despite better healthcare infrastructure, urban areas may also face barriers such as overburdened systems or socio-economic inequalities affecting vulnerable populations. This knowledge gap limits the ability to design targeted interventions that address the unique challenges urban and rural populations face. Understanding these disparities is crucial for policymakers, healthcare providers and stakeholders, as it provides the evidence needed to allocate resources effectively, design equitable health policies and implement strategies to improve maternal health outcomes across diverse settings. Addressing these gaps can contribute significantly to achieving Indonesia’s maternal health goals and aligning with global targets such as the SDGs. Therefore, this study aimed to identify the factors associated with the differences in the incompleteness of ANC coverage between urban and rural areas in Indonesia.

## Methods

### Data source

This study used data from Indonesian Basic Health Research 2018. Indonesian Basic Health Research is a community-based health study whose indicators can describe the national level up to the district level. It is conducted every 5 years by the Health Research and Development Agency. In this study, a descriptive cross-sectional survey was conducted employing quantitative research methods. We used two questionnaires: the Household Questionnaire and the Individual Questionnaire. These questionnaires are available at https://repository.badankebijakan.kemkes.go.id/id/eprint/4616/. The data set (RISKESDAS) can be accessed with approval from the Health Policy and Development Agency, Ministry of Health, Republic of Indonesia, at http://labmandat.litbang.kemkes.go.id/.

### Study population

The study population comprised women aged 15-49 years who had been pregnant in the last years during the interview, reaching as many as 190,604. The inclusion criteria were age of 15-49 years, previous pregnancy and ANC at least once during the last pregnancy. A total of 64,399 women were ultimately included. This study sample was divided into two subpopulations: 26,792 women living in urban areas and 37,607 women living in rural areas.

### Variables

This study adopted Andersen’s behavioural model of health service utilisation. Using this model^12^ provided a structured framework for exploring the complex interplay of factors influencing the incompleteness of ANC utilisation in Indonesia. Based on the model, 12 independent variables were included in this study: two variables from predisposing factors (educational level and employment status), four variables from enabling factors (ownership of health insurance, place of ANC services, travel time to health facilities and place of residence) and six variables from need factors (history of pregnancy, parity, history of abortion, multiple pregnancies, desired pregnancy and pregnancy complications). The study had only one dependent variable (incompleteness of ANC coverage).

This study divided ANC coverage into two categories: complete (four or more times) and incomplete (less than four times). The Indonesian government aims for at least four ANC visits per pregnancy. The type of place of residence was categorised into urban and rural; educational level into college, secondary education and primary education; employment status into working and not working; presence of health insurance into yes and no; ANC service setting into health facilities and home; travel time to health facilities into ≤15 min and >15 min; history of pregnancy into once and more than once; parity into ≤2 and >2; history of abortion into ever and never; multiple pregnancies into yes and no; desired pregnancy into yes and no; and pregnancy complications into yes and no. Pregnancy complications were defined as health problems or complaints experienced by the mother during pregnancy. The types of complications included persistent vomiting or persistent diarrhoea, hypertension, high fever, lack of foetal movement, bleeding in the birth canal, discharge of amniotic fluid, swollen legs with spasms, prolonged cough, chest pain/heart palpitations and others.

### Data analysis

Data cleaning and management were conducted using IBM SPSS Statistics for Windows, version 22 (IBM Corp., Armonk, N.Y., USA). Variables were recoded to meet the desired classification. The study employed univariate, bivariate and multivariate analyses. Univariate analysis was conducted to describe the distribution of each variable. Bivariate analysis was performed through the chi-square test to determine the association between the independent and dependent variables in urban and rural areas. Using logistic regression statistics, multivariate analysis was conducted to identify which independent variables have the most influence on the dependent variable.

## Results

Table 1 shows that incomplete ANC was more prevalent among the participants from rural areas (26.4%) than among those from urban areas (18.2%). Among the participants living in urban areas, 64.3% received secondary education, compared to 55.5% of those living in rural areas. Employment rates were slightly higher among the rural participants, with 42.3% employed compared to 39.6% among the urban participants. In terms of health insurance, 41.8% and 36.1% of the urban and rural participants were insured, respectively. Almost all participants accessed ANC at health facilities, with 99.6% in urban areas and 98.6% in rural areas. The travel time to health facilities differed significantly, with 58.8% of the urban participants requiring ≤15 min compared to 80.7% of the rural participants requiring >15 min. Regarding reproductive history, 31.0% and 32.9% of the urban and rural participants had been pregnant once, respectively. The proportion of women with one or two pregnancies was similar, at 74.0% in urban areas and 73.2% in rural areas. A history of abortion was reported by 15.2% of the urban participants and 13.4% of the rural participants. Multiple pregnancies were rare in both groups, occurring in 0.8% of the urban participants and 0.7% of the rural participants. Pregnancy intention and complications also differed. Desired pregnancies were reported by 90.1% of the urban participants and 93.3% of the rural participants. Pregnancy complications were slightly more common among the urban participants (25.8%) than among the rural participants (23.6%).

**Table 1:** Participant characteristics (N=64,399).

Variable	N	Place of residence	
		Urban (%)	Rural (%)
Complete	49,615	81.8	73.6
Incomplete	14,784	18.2	26.4
**Educational level**			
College	7253	16.2	7.7
Secondary education	38,106	64.3	55.5
Primary education	19,040	19.5	36.8
**Employment status**			
Working	26,493	39.6	42.3
Not working	37,906	60.4	57.7
**Ownership of health insurance**			
Yes	24,765	41.8	36.1
No	39,634	58.2	63.9
**Place of ANC services**			
Health facilities	63,769	99.6	98.6
Home	630	0.4	1.4
**Travel time to health facilities**			
≤15 min	23,017	58.8	19.3
>15 min	41,382	41.2	80.7
**History of pregnancy**			
Once	20,650	31.0	32.9
More than once	43,749	69.0	67.1
**Parity**			
≤2	47,328	74.0	73.2
>2	17,071	26.0	26.8
**History of abortion**			
Ever	9110	15.2	13.4
Never	55,289	84.8	86.6
**Multiple pregnancies**			
Yes	499	0.8	0.7
No	63,900	99.2	99.3
**Desired pregnancy**			
Yes	59,229	90.1	93.3
No	5170	9.9	6.7
**Pregnancy complications**			
Yes	15,786	25.8	23.6
No	48,613	74.2	76.4

Table 2 presents the results of the bivariate analysis using the chi-square test. In urban areas, there was a significant association of ANC coverage with educational level, employment status, place of ANC services, travel time to health facilities, history of pregnancy, parity and desired pregnancy (P<0.05). In contrast, ownership of health insurance, history of abortion, multiple pregnancies and pregnancy complications did not demonstrate a significant association with ANC coverage (P>0.05). In rural areas, educational level, ownership of health insurance, place of ANC services, travel time to health facilities, history of pregnancy, parity, multiple pregnancies, desired pregnancy and pregnancy complications exhibited a significant association with ANC coverage (P<0.05). Employment status, history of abortion and multiple pregnancies were not significantly associated with ANC coverage (P>0.05).

**Table 2:** Bivariate analysis of antenatal care coverage between urban and rural areas.

Variable	Urban			Rural		
	Incomplete	Complete	P	Incomplete	Complete	P
**Educational level**						
College	480 (11.1)	3861 (88.9)	*Ref.*	509 (17.5)	2403 (82.5)	*Ref.*
Secondary education	3054 (17.7)	14,177 (82.3)	0.000	4965 (23.8)	15,910 (76.2)	0.000
Primary education	1329 (25.5)	3891 (74.5)	0.000	4447 (32.2)	9373 (67.8)	0.000
**Employment status**						
Working	1736 (16.4)	8863 (83.6)	*Ref.*	4189 (26.4)	11,705 (73.6)	*Ref.*
Not working	3127 (19.3)	13,066 (80.7)	0.000	5732 (26.4)	15,981 (73.6)	0.949
**Ownership of health insurance**						
Yes	2048 (18.3)	9154 (81.7)	*Ref.*	3089 (22.8)	10,474 (77.2)	*Ref.*
No	2815 (18.1)	12,775 (81.9)	0.736	6831 (28.4)	17,212 (71.6)	0.000
**Place of ANC services**						
Health facilities	4827 (18.1)	21,856 (81.9)	*Ref.*	9702 (26.2)	27,384 (73.8)	*Ref.*
Home	36 (33.0)	73 (67.0)	0.001	219 (42.0)	302 (58.0)	0.000
**Travel time to health facilities**						
≤15 min	2655 (16.9)	13,096 (83.1)	*Ref.*	1406 (19.4)	5860 (80.6)	*Ref.*
>15 min	2208 (20.0)	8833 (80.0)	0.000	8515 (28.1)	21,826 (71.9)	0.000
**History of pregnancy**						
Once	1310 (15.8)	6985 (84.2)	*Ref.*	2799 (22.7)	9556 (77.3)	*Ref.*
More than once	3553 (19.2)	14,944 (80.8)	0.000	7122 (28.2)	18,130 (71.8)	0.000
**Parity**						
≤2	3101 (15.6)	16,716 (84.4)	*Ref.*	6403 (23.3)	21,108 (76.7)	*Ref.*
>2	1762 (25.3)	5213 (74.7)	0.000	3518 (34.8)	6578 (65.2)	0.000
**History of abortion**						
Ever	739 (18.1)	3339 (81.9)	*Ref.*	1292 (25.7)	3740 (74.3)	Rf
Never	4124 (18.2)	18,590 (81.8)	0.972	8629 (26.5)	23,946 (73.5)	0.348
**Multiple pregnancies**						
Yes	50 (22.5)	172 (77.5)	*Ref.*	63 (22.7)	214 (77.3)	*Ref.*
No	4813 (18.1)	21,757 (81.9)	0.219	9858 (26.4)	27,471 (73.6)	0.244
**Desired pregnancy**						
Yes	4065 (16.8)	20,080 (83.2)	Rf	8898 (25.4)	26,186 (74.6)	Rf
No	798 (30.2)	1849 (69.8)	0.000	1023 (40.6)	1500 (59.4)	0.000
**Pregnancy complications**						
Yes	1153 (16.7)	5763 (83.3)	Rf	2065 (23.3)	6805 (76.7)	Rf
No	3710 (18.7)	16,166 (81.3)	0.013	7856 (27.3)	20,881 (72.7)	0.000

Table 3 shows the differences in the association of the independent variables with the incompleteness of ANC between the urban and rural participants. Secondary and primary education, lack of health insurance, home as the ANC setting, parity greater than 2, travel time to health facilities exceeding 15 min, absence of a history of abortion, undesired pregnancy and absence of pregnancy complications were associated with incomplete ANC in rural areas. Conversely, secondary and primary education, home as the ANC setting, travel time to health facilities exceeding 15 min, parity greater than 2 and undesired pregnancy were associated with incomplete ANC in urban areas. In both rural and urban areas, incomplete ANC was related to secondary and primary education, home as the ANC setting, travel time to health facilities exceeding 15 min, parity greater than 2 and undesired pregnancy.

**Table 3:** Model of the urban-rural differences in the incompleteness of antenatal care coverage in Indonesia.

Variable	Urban			Rural		
	Model 1	Model 2	Model 3	Model 1	Model 2	Model 3
	OR (95% CI)	OR (95% CI)	OR (95% CI)	OR (95% CI)	OR (95% CI)	OR (95% CI)
**Educational level**						
College (ref.)						
Secondary education	1.595 (1.403-1.813)^*^	1.593 (1.402-1.811)^*^	1.631 (1.438-1.848)^*^	1.291 (1.151-1.509)^*^	1.292 (1.152-1.448)^*^	1.279 (1.143-1.430)^*^
Primary education	2.277 (1.959-2.647)^*^	2.273 (1.956-2.642)^*^	2.319 (2.002-2.685)^*^	1.700 (1.509-1.915)^*^	1.704 (1.514-1.919)^*^	1.688 (1.503-1.895)^*^
**Employment status**						
Working (ref.)						
Not working	1.079 (-0.986-1.181)	1.079 (0.986-1.181)		0.974 (0.915-1.037	0.974 (0.915-1.036)	0.244
**Ownership of health insurance**						
Yes (ref.)						
No	0.983 (0.902-1.071)			1.258 (1.182-1.340)^*^	1.258 (1.182-1.340)^*^	1.259 (1.182-1.340)^*^
**Place of antenatal care**						
Health facilities (ref.)						
Home	1.904 (1.199-3.023)^*^	1.900 (1.198-3.014)^*^	1.893 (1.193-3.004)^*^	1.881 (1.437-2.403)^*^	1.881 (1.473-2.403)^*^	1.880 (1.472-2.402)^*^
**Travel time to health facilities**						
≤15 min (ref.)						
>15 min	1.098 (1.002-1.204)^*^	1.098 (1.002-1.203)^*^	1.105 (1.008-1.211)^*^	1.491 (1.366-1.628)^*^	1.491 (1.266-1.627)^*^	1.491 (1.366-1.627)^*^
**History of pregnancy**						
Once (ref.)						
More than once	0.912 (0.812-1.023)	0.912 (0.812-1.024)		1.024 (0.946-1.108)	1.491 (1.266-1.627)^*^	1.491 (1.366-1.627)^*^
**Parity**						
≤2 (ref.)						
>2	1.575 (1.424-1.742)^*^	1.576 (1.425-1.743)^*^	1.513 (1.380-1.659)^*^	1.484 (1.384-1.591)^*^	1.498 (1.405-1.597)^*^	1.499 (1.406-1.598)^*^
**History of abortion**						
Ever (ref.)						
Never	1.063 (0.940-1.203)	1.062 (0.939-1.202)		1.109 (1.014-1.212)^*^	1.101 (1.010-1.201)^*^	1.100 (1.008-1.199)^*^
**Multiple pregnancies**						
Yes (ref.)						
No	0.848 (0.548-1.313)	0.845 (0.546-1.309)		1.264 (0.913-1.751)	1.264 (0.913-1.750)	1.100 (1.008-1.199)^*^
**Desired pregnancy**						
Yes (ref.)						
No	1.765 (1.555-2.003)^*^	1.766 (1.556-2.004)^*^	1.738 (1.532-1.971)^*^	1.629 (1.454-1.825)^*^	1.632 (1.457-1.828)^*^	1.629 (1.454-1.825)^*^
**Pregnancy complications**						
Yes (ref.)						
No	1.092 (0.985-1.212)	1.092 (0.984-1.210)		1.189 (1.105-1.279)^*^	1.189 (1.106-1.279)^*^	1.191 (1.107-1.281)^*^
**Total R^2^**			0.041			0.045

* Significant at P<0.05

OR: odds ratio; CI: Confidence Interval

Table 3 presents the results of the three models from the multivariate analysis, demonstrating the consistency of significant variables across all models. These models were constructed to confirm the robustness of the findings, with each model incorporating slight variations in covariate adjustments. The consistency observed showed the reliability of the predictors associated with incomplete ANC.

## Discussion

This study analysed the urban-rural differences in the incompleteness of ANC coverage in urban and rural areas of Indonesia. We found that 18.2% and 26.4% of the women in urban and rural areas received incomplete ANC, respectively. The proportion of women with incomplete ANC was larger in rural areas than in urban areas. This finding is in line with that of a study conducted in Ethiopia, wherein women from rural areas were 1.622 times more likely to receive incomplete ANC than their urban counterparts.^13^ In rural Bangladesh, only 25% of women were reported to attend the recommended four ANC visits, with a mere 11% initiating care in the first trimester.^14^

Various socio-economic, educational and systemic barriers influence the incompleteness of ANC coverage. The present study showed that primary education was associated with the incompleteness of ANC coverage in both urban and rural areas, consistent with previous reports indicating that women with lower educational attainment were less likely to attend ANC services regularly. For instance, primary education has been correlated with a higher likelihood of incomplete visits.^15,16^ Women with lower educational attainment are less likely to utilise ANC services, as seen in studies from Brazil and India, where education significantly influences care access.^17^ This finding may be attributed to the fact that education correlates with awareness of the importance of prenatal care.^14,15^

In our study, a lack of health insurance was associated with the incompleteness of ANC coverage in rural areas. A previous study in Kenya showed that a significant proportion of women received incomplete ANC packages, often due to late presentation and lack of insurance coverage.^18^ A study performed in Nigeria revealed that only 10.4% of rural women had eight or more ANC visits, with health insurance being a critical factor influencing this low utilisation.^19^ Insured women are 1.394 times more likely to receive complete ANC than uninsured women, highlighting the role of insurance in facilitating access to necessary healthcare services.^20^

Home as the ANC setting was found to be related to incomplete ANC in both urban and rural areas in this study. Financial constraints significantly influence the preference for home delivery and incomplete ANC visits.^21,22^ In South India, only 87.4% of women receive home visits, and many need to receive adequate prenatal advice.^23^ Many women face difficulties registering for health insurance due to costs, limiting their access to ANC services.^24^

Our findings showed that a parity greater than 2 was associated with the incompleteness of ANC coverage in both urban and rural areas. In a previous study in rural Uganda, 73% of high-parity women attended their first ANC visit late, often after 12 weeks of gestation. This suggests that higher parity is associated with incomplete ANC coverage, influenced by factors such as distance to health facilities.^25^ Women with unintended pregnancies are more likely to engage in unhealthy antenatal behaviours and are less likely to complete the recommended number of ANC visits.^26^

In the current study, an undesired pregnancy was related to incomplete ANC coverage in both rural and urban areas. Pregnancy intention plays a significant role in how women perceive the need for ANC services. Women with unintended pregnancies might feel less motivated to engage in routine prenatal visits, viewing them as less essential since their pregnancy is unplanned or not eagerly anticipated. This can lead to lower utilisation of ANC services, as they may need to thoroughly recognise or prioritise the benefits of regular checkups.

Our study also found that the absence of a history of abortion and pregnancy complications was associated with the incompleteness of ANC coverage in rural areas. Women without prior complications or a history of abortion may perceive their pregnancy as low-risk, potentially leading them to believe that regular ANC visits are unnecessary. This perception of safety can decrease their motivation to attend all recommended visits, as they may feel that these visits are only necessary for high-risk pregnancies. In Ghana, a lack of access to ANC increased home delivery odds by 27 times, highlighting the critical role of ANC in promoting facility-based births.^27^

Long travel times to health facilities significantly hinder ANC coverage, particularly in low-resource settings. In this study, a travel time to health facilities exceeding 15 min was associated with incomplete ANC. Other studies have shown that for every additional 10 min of travel time, women are more likely to arrive late or cancel appointments, which can lead to incomplete ANC attendance.^28^ Travel time is a critical barrier; community-based interventions and health system improvements can enhance ANC coverage, suggesting that targeted strategies may mitigate the effects of geographical inaccessibility.^29^ Geographic access to health facilities is crucial. In Ethiopia, the absence of a health facility within a 30-min distance was associated with a higher likelihood of home delivery.^30^

The disparities in ANC service utilisation between urban and rural areas are significant, with rural women experiencing notably lower access. To improve ANC coverage in rural areas, policy initiatives should focus on reducing structural and systemic barriers such as long travel times and inadequate healthcare infrastructures. Strengthening primary healthcare services and ensuring that ANC is universally accessible can help close the gap between urban and rural populations.

This study’s strengths lie in its use of a large, nationally representative data set and a systematic framework (Andersen’s behavioural model) to analyse the urban-rural disparities in ANC coverage. In contrast, the limitation of the study is its cross-sectional design. This design provided an overview of associations but limited the ability to infer causal relationships between the independent and dependent variables. There may be other unmeasured factors influencing ANC coverage, such as cultural beliefs, access to transportation or quality of care, which should have been included in the study but could affect the findings.

## Conclusion

This study highlights significant disparities in ANC coverage between urban and rural areas in Indonesia, with rural women exhibiting a higher prevalence of incomplete ANC. Key factors associated with incomplete ANC in rural areas include a lack of health insurance, the absence of abortion history and uncomplicated pregnancies. In urban areas, incomplete ANC is linked to a lower educational level, home as the ANC setting, longer travel times to health facilities, higher parity and unplanned pregnancies. Targeted interventions addressing these specific factors in both urban and rural settings are essential to improve ANC coverage and reduce maternal mortality in Indonesia.

## Data sharing statement

The raw data used in this study are available upon request to the corresponding author.

**How does this paper make a difference in general practice?**

- The study highlights significant disparities in antenatal care (ANC) coverage between urban and rural areas in Indonesia, with rural women facing higher rates of incomplete care.
- Utilising Andersen’s behavioural model, the study explores the interplay of predisposing, enabling and need factors affecting ANC utilisation.
- The findings emphasise the importance of health insurance programmes, education and improved access to healthcare facilities as critical measures to enhance ANC coverage.
